# Barbers' knowledge and practice about occupational biological hazards was low in Gondar town, North West Ethiopia

**DOI:** 10.1186/1471-2458-12-942

**Published:** 2012-11-01

**Authors:** Teresa Kisi Beyen, Ketema Tafess Tulu, Abdella Amano Abdo, Abera Shibru Tulu

**Affiliations:** 1 Department of Epidemiology and Biostatistics, Institute of public health, College Of Medicine and Health Science, University of Gondar, Gondar, Ethiopia; 2 Department of Biomedical Science, School of health and Hospital, Adama Science and Technology University, Assella, Ethiopia; 3 Department of Midwifery, College of Medicine and Health Science, University of Gondar, Gondar, Ethiopia; 4 Department of Environmental and Occupational health and safety, Institute of public health, College Of Medicine and Health Science, University of Gondar, Gondar, Ethiopia

## Abstract

**Background:**

Several health hazards including communicable diseases and skin conditions are associated with Barbers’ profession to which their visitors are exposed. Thus, knowledge and practice of Barbers would play a vital part in prevention and control of these health hazards. So, the aim of this study is to assess knowledge and practice, and associated factors among barbers about biological hazards associated with their profession in Gondar town, North West Ethiopia.

**Methods:**

To assess knowledge and practice, and associated factors among barbers about biological hazards associated with their profession in Gondar town, North West Ethiopia, A work place based cross-sectional study was conducted from March 28 to April 6, 2012. The total numbers of Barbers in the town were 960 of which 400 Barbers were participated in the study. Sample size was determined using the formula for single population proportion by considering, 51% proportion, knowledgeable Barbers from Jimma, Ethiopia, 95% level of confidence, 5% margin of error and 15% none response rate. The numbers of barbers included in the study were selected by using systematic random sampling. Data was collected by face to face interview using a structured and pre-tested questionnaire. Binary and multivariate logistic regression analyses were conducted to identify factors associated with knowledge and practice of barbers.

**Results:**

Of 400 barbers, only 72 (18%) had good knowledge about biological hazards associated to their profession, While only 61 (15.3%) were practicing safely during barbering. Knowledge of the barbers was associated significantly with educational level, owner of the business, working hour and work experience, while practice was associated only with availability of UV sterilizers in the room and working hour.

**Conclusion:**

Barbers’ practice and knowledge to prevent biological hazards associated with their profession is very poor. Thus, giving training for the Barbers is required toward prevention of biological hazards associated to their profession.

## Background

The word ‘Barber’ originates from the Latin word. Barber meaning ‘beard’. A Barber is a person whose occupation is to cut any type of hair, give shaves, and trim beards. They are important professionals of the community which are owned, cared and financed by the community. It is the need of their profession to utilize instruments like clips, blades etc. [1].

Several health hazards including communicable diseases and skin conditions are associated with Barbers’ profession to which their visitors are exposed. The diseases of primary importance linked to this profession are ringworm disease, (through direct contact), infestation of head louse, staphylococcal, streptococcus, Scabies (through contaminated towels, combs, and aprons) and Hepatitis B, hepatitis C, tetanus and AIDS (contaminated blades and clips) [2].

It is reported from Pakistan, Japan, Egypt, Israel, USA, and Italy that HCV, and from Bangladesh, Pakistan, India, Iran, Israel, Italy that HBV can be transferred by blade sharing and barber-related instruments [1,3-10].

The other study conducted in Rawalpindi and Islamabad, the capital twin cities of Pakistan revealed that 39.6% of barbers knew that hepatitis B and C are viral diseases, 26.6% of barbers knew that it can lead to cancer, 34.6% have heard advertisements about hepatitis. Only 9.8% of barbers had taken the HBV vaccine, and most people thought that allopathic therapy is the best treatment option for hepatitis [11].

According to study conducted in Janjua and Nizamy 12.5% of barbers knew that hepatitis B and C are diseases of the liver causing hepatitis, 63.5% changed the blade for every customer, 7.2% knew that HBV can be prevented by vaccination, 18.7% were sterilizing the instruments, and 100% of them were disposing blades in sewerage waste [12].

The study conducted in Izmir, Turkey revealed that 41.2% of barbers reported that they used gloves and 15.2% had used protective clothing within the last month. It was determined that 4.4% practiced dry air sterilization and 3.4% allowed their customers to use their own equipment. The vast majority (80.9%) reported that they used an ultraviolet sterilization device whereas others preferred to wash their equipment with soapy water (9.2%), wipe it with alcohol (41.7%), or to immerse it in a disinfectant chemical (12.7%). Only one-third of these employees washed their hands before and after each process [13].

Many studies revealed that there was significant difference in level of awareness among barbers in respect of age, educational status, Work load, accessibility to media (like TV) and work experience. [1,3,11,13,14].

A large proportion of population is enjoying the services of Barbers in our community and their place of work and profession may be a potential source of infectious diseases transmission silently in the community. The study done in Jimma, Ethiopia on knowledge of Barbers about HIV/AIDS revealed that, only 51% of the respondents knew the possible transmission of HIV during their practice [15].

Considering the grave consequences of infections especially Hepatitis B, C and AIDS, associated with this profession; awareness about these health hazards among barbers would play a vital part in prevention and control of these infections [2].

It is essential and urgent to promote awareness of these risks among all, especially the public authorities, and to formally ban Barbers from illegal practice. Although it will be a huge task for public health practitioners to bring about the behavioral change towards unhealthy practices, they have to accept this challenge in order to protect the community at large. Thus, the aim of this study is to assess knowledge and practice, and associated factors among barbers about health hazards associated with their profession in Gondar town, North West Ethiopia.

## Methods

To assess knowledge and practice, and associated factors among barbers about biological hazards associated with their profession in Gondar town, North West Ethiopia, A work place based cross-sectional study was conducted from March 28 to April 6, 2012. Gondar town is the capital of North Gondar zone. It is one of the historical towns in the country and located at 727km Northwest of Addis Ababa (capital of the country). According to the 2007 Ethiopian census report, Gondar has a total population of 206, 987 and more than half (108,902) of them are females. Administratively the town is divided into 12 administrative areas. The town had about 320 barber houses which had averagely about three Barber professionals. The total numbers of Barbers in the town were 960 of which 400 Barbers were participated in the study. Sample size was determined using the formula for single population proportion by considering, 51% proportion, knowledgeable Barbers from Jimma, Ethiopia, 95% level of confidence, 5% margin of error and 15% none response rate. Codes were given for Barbers in the town. The numbers of barbers included in the study were selected by using systematic random sampling.

Data on socio demographic characteristics, Environmental factors and Health hazards (HIV/AIDs, HBV, HCV, Staphylococcus, Streptococcus, Ringworm and Head lice ) related to their profession were collected by face to face interview using a structured and pre-tested questionnaire, first prepared in English and translated to Amharic which was developed from literature and standards of Barbers. Question related to the etiology, transmission mechanism, prevention mechanism and potential instruments to transmit each health hazards have been asked. Two supervisors and six trained data collectors were participated in the data collection process.

The collected data were entered, cleaned and edited using EPI INFO 2011 statistical software and then exported to SPSS version 20 for further analysis. Descriptive statistics of the collected data were done for most variables in the study using statistical measurements. Frequency tables, graphs, percentages, means and standard deviations were used. Individual who scored fifty percent and above for the questions related to knowledge were categorized under good knowledge. Bivariate analysis was conducted primarily to check which variables have association with the dependent variables individually. Variables found to have association with the dependent variables at 0.2 probability were then entered in to multivariate logistic regression for controlling the possible effect of confounders and finally the variables which had significant association were identified on the basis of OR, with 95%CI and 0.05 p-values and fitted into the final model.

The study was conducted after getting permission from the ethical review board of university of Gondar. Then, an informed consent was obtained from each study participants to participate in the study. Those barbers who refused to participate in the study were not forced. Each respondent was informed about the objective of the study. Confidentiality was granted for information collected by keeping the privacy of the respondents during interview.

## Results

### Socio-demographic characteristics of barbers

Out of total sample size (442), 400 (90.5%) Barbers responded with non response rate of 9.5%. Out of the total respondents 372 (93%) were males. About 144 (36%) of the respondents were married. Respondents’ age ranged from 20 to 52 years with mean (standard deviation) of 26.35 (±4.93) years. The daily mean (standard deviation) income of the respondents was 37.07 (±15.56) birr. The average numbers of barbers per house were three. All of the study participants responded that they had radio in their working room and no any standards to open the barbering service. (Table 1)

**Table 1:** Socio demographic characteristics of Barbers in Gondar town, April, 2012, (n =400)

**Variables**		**n (%)**
Sex	Male	372 (93)
	Female	28 ( [7])
Religion	Orthodox	312 (78)
	Muslim	88 (22)
Educational level	12 complete and above	244 (61)
	Secondary [9-12]	88 (22)
	Primary [1-8]	68 ( [17])
Marital status	Single	256 (64)
	Married	144 (36)
Working hours per day	≤8 hours	296 (74)
	>8 hours	104 (26)
Work experience	≤5 years	236 (59)
	>5 years	164 (41)
Number of barbers per room	Only one	172 (43)
	>1	228 (57)
Owner of the business	My own	132 (33)
	Shared	192 (48)
	Employed by others	76 ( [19])
TV access	Yes	52 ( [13])
	No	348 (87)
Towel sterilizer access	Yes	68 ( [17])
	No	332 (83)
UV sterilizer access	Yes	184 (46)
	No	216 (54)
Took training	Yes	39 ( [9])
	No	364 (91)

### Knowledge level of the barbers

Of total study participants 312 (78%) responded that they heard about biological hazards related to their profession, of which 196 (63%) heard from TV, 36 (12%) from training related to health and safety, 204 (66%) by reading book and 184 (59%) by reading newsletters related to health and safety. (Table 2)

**Table 2:** Source of information for Barbers who heard about biological hazards related to their work, in Gondar town, April, 2012. (n=312)

**Variables**	**Yes, n (%)**	**No, n (%)**
From TV	196 (63)	116 (37)
From training	36 ( [12])	276 (88)
By reading book	204 (66)	108 (34)
By reading newsletters	184 (59)	128 (41)

### Knowledge of barbers about each biological hazard related to their work

Based on the knowledge questions prepared for each biological hazards all of the respondents had good knowledge about HIV/AIDS and head lice while all of them had poor knowledge about staphylococcus and streptococcus. Of the total respondents 332 (80%), 45 (11%) and 45 (11%) had good knowledge about ringworm, HBV and HCV respectively. (Table 3)

**Table 3:** Knowledge of barbers about each biological hazards related to their work, April, 2012, (n=400)

**Health hazards**	**Good knowledge, n (%)**	**Poor knowledge, n (%)**
HIV/AIDs	400 **(100)**	0
HBV	45 [11]	355 (89)
HCV	45 [11]	355 (89)
Staphylococcus	0	400 (100)
Streptococcus	0	400 (100)
Ringworm	332 **(80)**	68 (20)
Head lice	300 **(75)**	100 (25)

**Figure 1:**
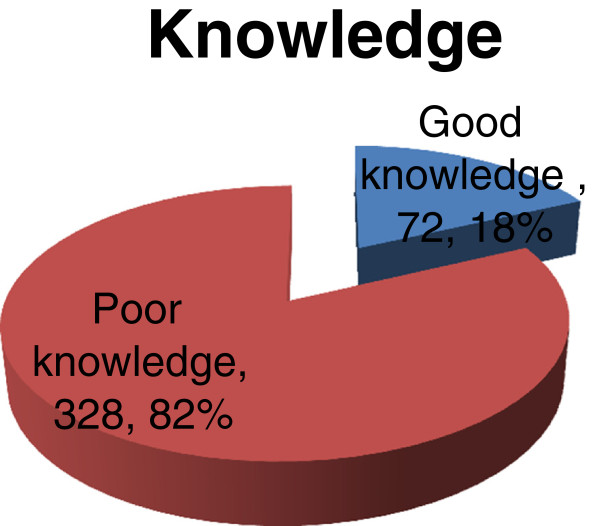
Knowledge of barbers about biological hazards associated to their work in Gondar town, April, 2012, (n=400).

Based on knowledge related questions, of 400 barbers, only 72 (18%) had good knowledge about biological hazards related to their work. (Figure 1)

### Practice of the barbers towards prevention of biological hazards related to their work

Out of total Barbers 284 (71%) responded that they did not wash hands for new customers while 232 (58%), 260 (65%), 376 (94%), 392 (98%), 376 (94%) and 385 (97%) neither changed nor sterilized razors, shavers, scissors, Brushes, Combs and towels during giving barbering service to different customers respectively. Of the total study participants 392 (98%) neither washed nor changed apron for new customers during barbering. (Table 4)

**Table 4:** Practice of Barbers towards prevention of biological hazards related to their work, April, 2012, (n = 400)

**Practice of barbers**	**Yes, n (%)**	**No, n (%)**
Wash hands for new customers	116 (39)	**284 (71)**
Use neither changed nor sterilized razors	**232 (58)**	168 (42)
Use neither changed nor sterilized shavers	**265 (65)**	135 (35)
Use neither changed nor sterilized scissors	**376 (94)**	24 [6]
Use neither changed nor sterilized brushes	**392 (98)**	6 [2]
Use neither changed nor sterilized combs	**376 (94)**	24 [6]
Use neither changed nor sterilized towels	**385 (97)**	15 [3]
Use neither changed nor washed apron	**392 (98)**	8 [2]

**Figure 2:**
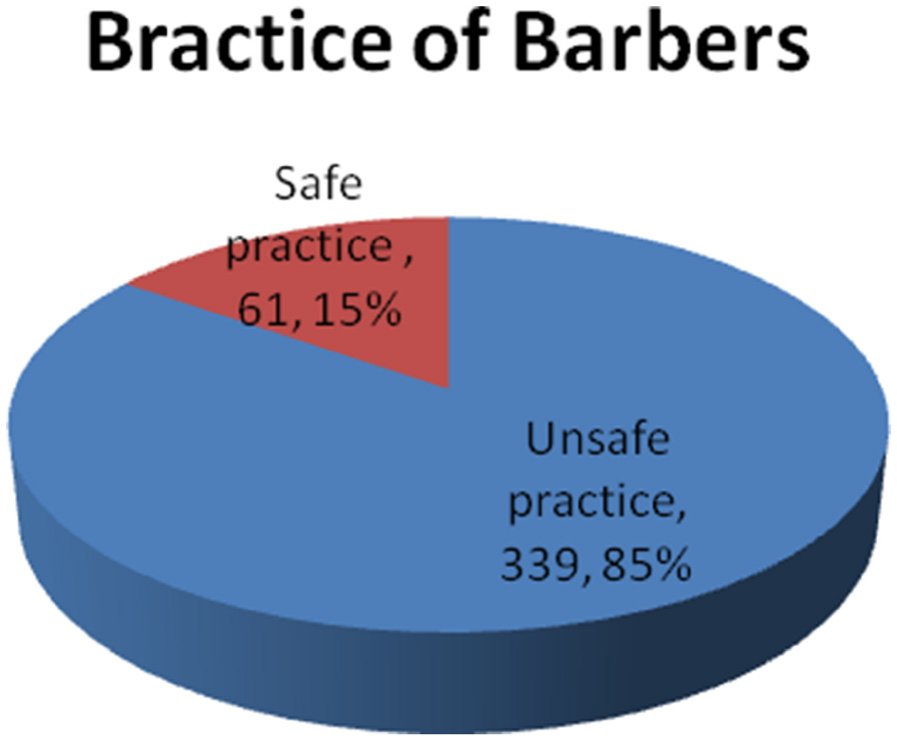
General practice of Barbers towards prevention of biological hazards related to their work in Gondar town, April, 2012, (n=400).

Generally, out of the total study participants only 61 (15.3%) were practicing safely during giving barbering service to different customers based on the questions prepared for practice. (Figure 2)

### Factors associated with knowledge of the barbers

Socio demographic and environmental factors were analyzed in relation to Barbers’ knowledge by bivariate and multivariate logistic regression analyses model. In the bivariate logistic regression analysis, Knowledge of the Barbers was associated significantly with educational level, owners of the business, working hour, work experience, availability of UV sterilizer in the room and number of barbers per room. However, in the multivariate logistic regression analysis, knowledge of the barbers was associated significantly with educational level, owner of the business, working hour and work experience. (Table 5)

**Table 5:** Factors associated with barbers’ knowledge of biological hazards related to their work in Gondar town, April, 2012 (n=400)

**Variables**	**Knowledge**					
		**Pood, n**	**Poor, n**	**Crude OR (95% CI)**	**Adjusted OR (95%CI)**	**p-value**
Education	12 complete and above	44	171	5.98(2.08,17.16)	3.25 (1.59,6.64)	0.001**
	Secondary	24	64	8.72(2.89,26.33)	3.02 (1.01,9.52)	0.048**
	Primary	4	93	1		
Owner of the business	My Own	12	120	1		
	Shared	36	156	2.31(1.10,4.92)	1.96 (1.02,4.44)	0.04**
	Employed by others	24	52	4.62(2.02,10.67)	4.41 (1.71,11.39)	0.002**
Working hour	≤8hour	46	148	2.33(1.32,4.14)	2.11 (1.80,5.75)	0.000**
	>8hour	24	180	1		
Work experience	≤5 years	21	176	1		
	>5 years	51	152	2.81(1.57,5.08)	2.50 (2.33,9.23)	0.000**
Availability of UV sterilizer	Yes	24	173	0.45 (0.25,0.79)*		
	No	48	155	1		
Number of barbers per room	Only one	40	132	1.86 (1.08,3.21)*		
	>1	32	196	1		

* Associated only by univariate analysis, ** Associated by both univariate and multivariate, at p-value ≤ 0.05.

### Factors associated with practice of the barbers

Socio demographic and environmental factors were analyzed in relation to Barbers’ practice towards prevention of health hazards related to their work by bivariate and multivariate logistic regression analyses model. In the bivariate logistic regression analysis, practice of the Barbers was associated significantly with availability of UV sterilizers in the room, Availability of towel sterilizers in the room and working hour. However, in the multivariate logistic regression analysis, practice of barbers was only associated significantly with availability of UV sterilizers in the room and working hour. (Table 6)

**Table 6:** Factors associated with Barbers’ practice towards prevention of biological hazards related to their work in Gondar town, April, 2012, (n= 400)

**Variables**	**Practice**					
		**Safe, n**	**Unsafe, n**	**Crude OR (95% CI)**	**Adjusted OR (95%CI)**	**p-value**
Availability of UV sterilizer	Yes	44	204	3.91(2.07,7.46)	2.93(1.55,5.52)	0.001**
	No	17	135	1		
Availability of towel sterilizer	Yes	19	49	2.68(1.37,5.20)*		
	No	42	290	1		
Working hour	≤8	14	180	1	1	
	>8	47	159	3.85(1.96,7.69)	3.23(1.69,6.25)	0.000**

* Associated only by univariate analysis, ** Associated by both univariate and multivariate at p-value ≤ 0.05.

## Discussion

This study disclosed the knowledge and practice of barber on biological hazards associated to their work, and factors associated to knowledge and practice of barbers in Gondar town. Thus, out of the total study participants, only 72 (18%) had good knowledge about biological hazards related to their work, which is lower than the studies conducted in Jimma, Ethiopia 51% [15], Pakistan, Kharian city of district Gujrat 42% [1], Rawalpindi and Islamabad 39.6% [11], Bahra Kahu, Islamabad 38% [16], and Nigeria 24.8% [17]. One of the possible reasons causing difference in the knowledge level could be the number of health hazards covered to measure the knowledge of the barbers by the studies, when this study covers more health hazards than stated studies. However, it in line with the study conducted in Nigeria, Ibadan on knowledge of HIV (16.7%) [18].

In this study, educational level of Barbers showed a significant association with Barbers’ knowledge about biological hazards related their work. Barbers in the education level of 12 complete and above, and secondary (9-12grades) were more than three times more likely to have good knowledge about biological hazards related to their work when compared to those in the primary educational level [AOR=3.25, 95% CI: 1.59, 6.64] and [AOR=3.02, 95% CI: 1.01, 9.62] respectively. The result was consistent with the study conducted in Pakistan, Kharian city of Gujarat [1], Rawalpindi and Islamabad [11] and Nigeria, Ibadan [18] which suggests that barbers who had higher schooling were found to have better knowledge about health hazards related to barbering indirectly from their formal education.

Barbers who employed by others and opened barbering service by sharing were more than four and one point nine six times more likely to have knowledge about biological hazards related to their work when compared to those who had their own barbering service [AOR=4.41, 95% CI: 1.71, 11.39] and [AOR=1.96, 95% CI: 1.02, 4.44] respectively. The possible reason for the Barbers’ knowledge difference between these groups could be the educational level deference. The study showed that most Barbers employed by others and opened barbering services by sharing was in the higher education level.

Barbers who spent eight or less hour per day on work were more than two times more likely to have knowledge about biological hazards related to their work when compared to those who spent more than eight hour per day on work [AOR=2.11, 95% CI: 1.80, 5.75]. This result is in line with the study conducted in Pakistan, Kharian city of Gujrat [1]. The possible reasons could be barbers who spent less time on work had time for access to media and they were in the higher education level from which they may discern about health hazards associated to their work.

In this study barbers who had work experience more than five years were more than two times more likely to know biological hazards related to their work than those who had five or less work experience [AOR=2.50, 95% CI: 1.33, 5.23]. This result is supported by studies conducted in pakistan, Kharian city of Gujrat [1], which indicate, as the barbers experience had been more and more the exposure to the hazards increased which may increase their knowledge.

Based on the questionnaires prepared for the practice, the practice of barbers towards prevention of biological hazards related to their work were low. Accordingly out of the total study participants only 61 (15.3%) were practicing safely to prevent biological hazards related to their work during barbering service to different customers. This finding is lower than the studies conducted in Pakistan; Kharian city of district Gujrat [1], Bahra Kahu, Islamabad [16], Rawalpindi and Islamabad and Sana’a City, Yemen [19]. The possible reason for the deference could be, the stated studies used single practice to define the practice of the study participants when this study considers many activities of the barbers to define the barbers’ practice to prevent the health hazards related to their work. The other reason could the socio economic characteristic of the study participants which may affect the availability of the required materials for safe practice.

The study revealed that Barbers who had UV sterilizer in their room were more than two times more likely to practice safely during barbering service when compared to those who had not [AOR=2.93, 95% CI: 1.55, 5.52]. Similarly barbers those spent more than eight hour on work were more than three times more likely to practice safely to prevent biological hazards associated to their work during barbering service when compared to those spent eight or less hour on work [AOR=3.65, 95% CI: 1.69, 6.25]. The possible reasons for this could be Barbers who work more than eight hours per day got more income so that they can buy materials like UV and towel sterilizers which are important for practice to prevent health hazards related to barbers’ work.

There are, of course, limitations in this study, as it was depending on self reported data of the participants which was susceptible to social desirability bias causing under or overestimation. Similarly the study has not addressed the effect of attitude of the Barbers on knowledge and practice. Even though this study tried to address some important factors, the type of chemical disinfectants used and duration for sterilization were not addressed. In addition, the study was not out of the limitations of cross sectional study like identifying the temporal relationship.

## Conclusion

In conclusion, Barbers’ knowledge level about biological hazards associated with their profession is very poor. Majority of the Barbers practiced unsafely in barbering. Availability of UV sterilizers in the Barbers’ room and working hours had significant association with the practice of the Barbers while education, owner of the business, working hours and work experience had significant association with the knowledge of the Barbers. Thus, national occupational safety and health competent authorities should provide training for the Barbers on occupational biological hazards to increase the knowledge and practice of these workers on these health hazards.

## Competing interests

The authors declare that they have no competing interests.

## Authors' contributions

Teresa Kisi, wrote the proposal, participated in data collection, analyzed the data and drafted the paper. Ketema Tafess, Abdella Amano and Abera Shibru approved the proposal with some revisions, participated in data analysis and revised subsequent drafts of the paper. All authors read and approved the final manuscript.

## Pre-publication history

The pre-publication history for this paper can be accessed here:

http://www.biomedcentral.com/1471-2458/12/942/prepub
